# (3β,18β,20β)-*N*-Eth­oxy­carbonyl­methyl-3-nitrato-11-oxoolean-12-ene-29-carboxamide methanol monosolvate

**DOI:** 10.1107/S1600536812012561

**Published:** 2012-03-31

**Authors:** Laszlo Czollner, Ulrich Jordis, Kurt Mereiter

**Affiliations:** a Institute of Applied Synthetic Chemistry, Vienna University of Technology, Getreidemarkt 9/163, A-1060 Vienna, Austria; b Institute of Chemical Technologies and Analytics, Vienna University of Technology, Getreidemarkt 9/164SC, A-1060 Vienna, Austria

## Abstract

The title compound, C_34_H_52_N_2_O_7_·CH_4_O, is the methanol solvate of a difunctionalized derivative of the therapeutic agent 18β-glycyrrhetinic acid, a penta­cyclic triterpene. The five six-membered rings of the glycyrrhetinic acid moiety show normal geometries, with four rings in chair conformations and the unsaturated ring in a half-chair conformation. This moiety is substituted by a nitrate ester group and an *O*-ethyl­glycine group. In the crystal, the nonsolvent mol­ecules are packed parallel to (010) in a herringbone fashion with the nitrato, ethyl­glycine and methanol-O atom being proximate. The methanol solvent mol­ecule is anchored *via* a donated O—H⋯O_ac­yl_ and an accepted N—H⋯O hydrogen bond, giving rise to infinite zigzag chains of hydrogen bonds parallel to [100]. Two weak intermolecular C—H⋯O interactions to the methanol and to an acyl oxygen establish links along [100] and [010], respectively.

## Related literature

For overviews on the therapeutic aspects of glycyrrhetinic acid, see: Baran *et al.* (1974 ▶); Asl & Hosseinzadeh (2008 ▶). For the synthesis of new derivatives of 18β-glycyrrhetinic acid and their effect on 11β-hy­droxy­steroid dehydrogenase, see: Su *et al.* (2004 ▶); Beseda *et al.* (2010 ▶); Amer *et al.* (2010 ▶). For the crystal structure of 18β-glycyrrhetinic acid, see: Campsteyn *et al.* (1977 ▶); Alvarez-Larena *et al.* (2007 ▶). For the crystal structures of derivatives of 18β-glycyrrhetinic acid, see: Beseda *et al.* (2010 ▶); Amer *et al.* (2010 ▶); Czollner *et al.* (2011 ▶).
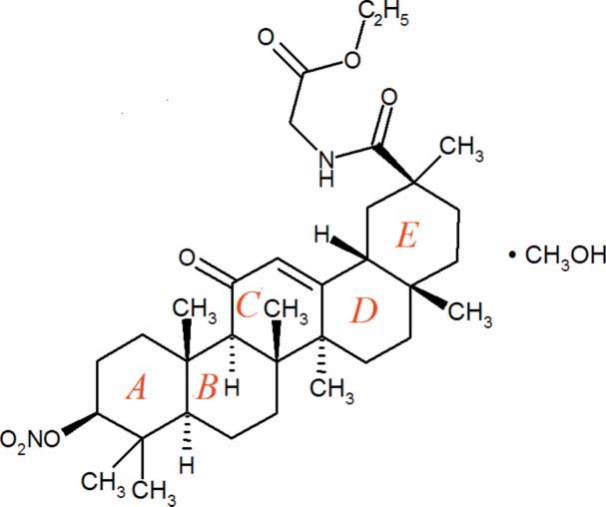


## Experimental

### 

#### Crystal data

C_34_H_52_N_2_O_7_·CH_4_O
*M* *_r_* = 632.82
Orthorhombic, 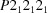

*a* = 10.1598 (8) Å
*b* = 11.1275 (9) Å
*c* = 30.387 (2) Å
*V* = 3435.3 (5) Å^3^
*Z* = 4
Mo *K*α radiation
μ = 0.09 mm^−1^
*T* = 100 K
0.55 × 0.53 × 0.15 mm

#### Data collection

Bruker Kappa APEXII CCD diffractometer
Absorption correction: multi-scan (*SADABS*; Bruker, 2008 ▶) *T*_min_ = 0.88, *T*_max_ = 1.00
49017 measured reflections
5565 independent reflections
5044 reflections with *I* > 2σ(*I*)
*R*_int_ = 0.036

#### Refinement

*R*[*F* ^2^ > 2σ(*F* ^2^)] = 0.044
*wR*(*F* ^2^) = 0.122
*S* = 1.10
5565 reflections
416 parameters
H-atom parameters constrained
Δρ_max_ = 0.68 e Å^−3^
Δρ_min_ = −0.38 e Å^−3^

### 

Data collection: *APEX2* (Bruker, 2008 ▶); cell refinement: *SAINT* (Bruker, 2008 ▶); data reduction: *SAINT*, *SADABS* and *XPREP* (Bruker, 2008 ▶); program(s) used to solve structure: *SHELXS97* (Sheldrick, 2008 ▶); program(s) used to refine structure: *SHELXL97* (Sheldrick, 2008 ▶); molecular graphics: *Mercury* (Macrae *et al.*, 2006 ▶); software used to prepare material for publication: *PLATON* (Spek, 2009 ▶) and *publCIF* (Westrip, 2010 ▶).

## Supplementary Material

Crystal structure: contains datablock(s) global, I. DOI: 10.1107/S1600536812012561/xu5490sup1.cif

Structure factors: contains datablock(s) I. DOI: 10.1107/S1600536812012561/xu5490Isup2.hkl

Additional supplementary materials:  crystallographic information; 3D view; checkCIF report

## Figures and Tables

**Table 1:** Hydrogen-bond geometry (Å, °)

*D*—H⋯*A*	*D*—H	H⋯*A*	*D*⋯*A*	*D*—H⋯*A*
N2—H2*N*⋯O8^i^	0.88	2.04	2.806 (3)	144
O8—H8⋯O5	0.84	1.89	2.728 (2)	177
C1—H1*A*⋯O4	0.99	2.34	2.968 (2)	120
C19—H19*B*⋯O8^i^	0.99	2.40	3.359 (3)	163
C25—H25*A*⋯O4	0.98	2.41	3.058 (3)	123
C34—H34*B*⋯O5^ii^	0.98	2.58	3.515 (4)	160

Symmetry codes: (i) 
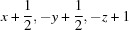
; (ii) 
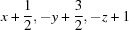
.

**Fig. 1.**
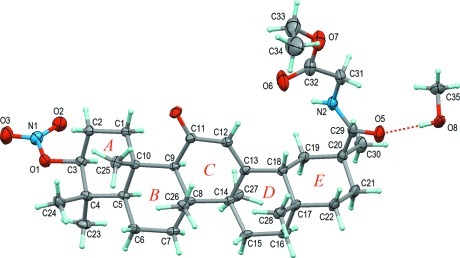
The asymmetric unit of (I), with displacement ellipsoids for the non-H atoms drawn at the 50% probability level. Red capitals are the ring designations.

**Fig. 2.**
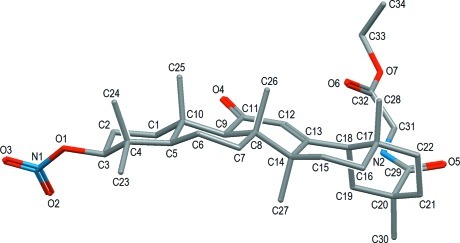
The molecular structure of (I) in a side-view showing the conformation of the rings more clearly. H atoms have been omitted for clarity.

**Fig. 3.**
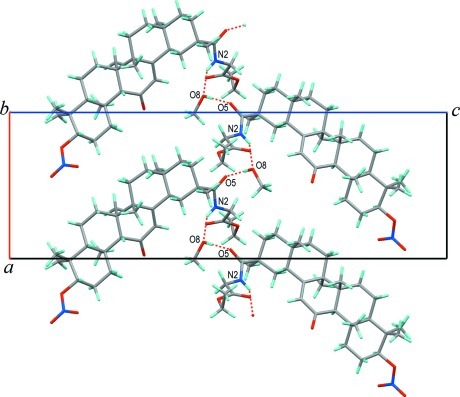
A section of the structure of (I), in a view down the b axis, showing the methanol–acyl O—H···O and amide–methanol N—H···O hydrogen bonds, as dashed red lines, forming a zigzag chain along [100].

## supplementary crystallographic information

### Comment

The title compound, (I), was synthesized within a research program (Beseda *et al.*, 2010; Amer *et al.*, 2010) designed to create new therapeutically useful derivatives of 18β-glycyrrhetinic acid (GA), an agent for the treatment of metabolic deseases (Baran *et al.*, 1974; Asl & Hosseinzadeh, 2008). For new therapeutic applications, GA is typically modified on ring A (C1 - C5 and C10), on ring C (C8 - C15), and/or on the terminal carboxyl group of C29 (Su *et al.*, 2004; Beseda *et al.*, 2010; Czollner *et al.*, 2011). In the title compound these modifications comprised the introduction of an *O*-ethylglycine group N-bonded to the COOH group of GA, and, as an uncommon feature, a nitrate ester group replacing the 3-hydroxy group of GA. The compound was then crystallized from methanol to give the stoichiometric crystalline methanol solvate (I). A view of the asymmetric unit is shown in Fig. 1. The GA fragment (C1 through C30, O1, O4, O5) features usual bond lengths, bond angles, and conformation (Campsteyn *et al.*, 1977; Alvarez-Larena *et al.*, 2007; Beseda *et al.*, 2010; Czollner *et al.*, 2011). There are four six-membered saturated carbocycles (A, B, D, and E) in chair and the unsaturated ring C in half-chair conformation (Fig. 2). The carboxamide group O5═C29—N2 is *endo*-oriented with respect to the amide nitrogen N2 (C19—C20—C29—N2 = -28.1 (3)°), in contrast to a propargyl amide derivative of GA, where it is *exo*-oriented (Czollner *et al.*, 2011; corresponding torsion angle 162.3°). In the crystal lattice of (I) the non-solvent molecules are arranged in undulating layers parallel to (010) and adopt a typical herring-bone pattern within these layers (Fig. 3). These layers repeat by 2_1_ axes parallel to [010]. The oxygen and nitrogen bearing ends of the GA molecules and the methanol solvent molecules are accumulated in reagions near *z*≈ 0, 1/2, and 1, and are crosslinked by O—H···O, N—H···O and C—H···O interactions (Table 1). The most prominent of them are the hydrogen bonds O8—H8···O5 and N2—H2n···O8^i^, which are donated and accepted by the methanol molecule. The methanol molecule and the carboxamide moiety O5═C29—N2 thereby build up an infinite zigzag hydrogen bond chain parallel to [100], as shown in Fig. 3. The nitrato group (N1, O1, O2, O3) is stereochemically inactive by showing no C,N,O—H···O interactions within the usual geometrical limits (Table 1; cut-off values are H···O ≤ 2.60 Å, *X*—H···O ≥ 120°).

### Experimental

To a stirred solution of acetic anhydride (5 ml) and concentrated nitric acid (2 ml) was added *N*-(ethoxycarbonyl-methyl)-3-hydroxy-11-oxo-olean-12-ene-29-carboxamide (555 mg, 1.0 mmol; compound 26f of Beseda *et al.*, 2010) at 273 K. After 30 min the reaction mixture was dropped to 200 ml of ice water. The solid product obtained was filtered, dried and recrystallized from 3 ml of dichloromethane and 5 ml of n-hexane to yield 400 mg (66.6%) of the desired product as colourless powder. An analytical sample of (I) was then obtained by recrystallization from methanol.

### Refinement

All H atoms were placed in calculated positions and thereafter treated as riding. A torsional parameter was refined for each methyl group. *U*_iso_(H) = 1.2*U*_eq_(C_non-methyl_) and *U*_iso_(H) = 1.5*U*_eq_(C_methyl_) were used. Because of insignificant anomalous dispersion effects, the 4435 Friedel pairs were merged prior to the final refinement. The absolute structure of the parent compound 18β-glycyrrhetinic acid is known.

### Crystal data

**Table:**

C_34_H_52_N_2_O_7_·CH_4_O	*F*(000) = 1376
*M**_r_* = 632.82	*D*_x_ = 1.224 Mg m^−^^3^
Orthorhombic, *P*2_1_2_1_2_1_	Mo *K*α radiation, λ = 0.71073 Å
Hall symbol: P 2ac 2ab	Cell parameters from 9832 reflections
*a* = 10.1598 (8) Å	θ = 2.4–30.4°
*b* = 11.1275 (9) Å	µ = 0.09 mm^−^^1^
*c* = 30.387 (2) Å	*T* = 100 K
*V* = 3435.3 (5) Å^3^	Plate, colourless
*Z* = 4	0.55 × 0.53 × 0.15 mm

### Data collection

**Table:**

Bruker Kappa APEXII CCD diffractometer	5565 independent reflections
Radiation source: fine-focus sealed tube	5044 reflections with *I* > 2σ(*I*)
Graphite monochromator	*R*_int_ = 0.036
φ and ω scans	θ_max_ = 30.0°, θ_min_ = 2.7°
Absorption correction: multi-scan (*SADABS*; Bruker, 2008)	*h* = −14→14
*T*_min_ = 0.88, *T*_max_ = 1.00	*k* = −15→15
49017 measured reflections	*l* = −42→42

### Refinement

**Table:**

Refinement on *F*^2^	Primary atom site location: structure-invariant direct methods
Least-squares matrix: full	Secondary atom site location: difference Fourier map
*R*[*F*^2^ > 2σ(*F*^2^)] = 0.044	Hydrogen site location: inferred from neighbouring sites
*wR*(*F*^2^) = 0.122	H-atom parameters constrained
*S* = 1.10	*w* = 1/[σ^2^(*F*_o_^2^) + (0.0659*P*)^2^ + 1.0649*P*] where *P* = (*F*_o_^2^ + 2*F*_c_^2^)/3
5565 reflections	(Δ/σ)_max_ < 0.001
416 parameters	Δρ_max_ = 0.68 e Å^−^^3^
0 restraints	Δρ_min_ = −0.38 e Å^−^^3^

### Special details

**Table:**

Geometry. All e.s.d.'s are estimated using the full covariance matrix. The cell e.s.d.'s are taken into account individually in the estimation of e.s.d.'s in distances, angles and torsion angles; correlations between e.s.d.'s in cell parameters are only used when they are defined by crystal symmetry.
Refinement. Refinement of *F*^2^ against ALL reflections. The weighted *R*-factor *wR* and goodness of fit *S* are based on *F*^2^, conventional *R*-factors *R* are based on *F*, with *F* set to zero for negative *F*^2^. The threshold expression of *F*^2^ > σ(*F*^2^) is used only for calculating *R*-factors(gt) *etc*. and is not relevant to the choice of reflections for refinement. *R*-factors based on *F*^2^ are statistically about twice as large as those based on *F*, and *R*- factors based on ALL data will be even larger.

### Fractional atomic coordinates and isotropic or equivalent isotropic displacement parameters (Å^2^)

**Table:**

	*x*	*y*	*z*	*U*_iso_*/*U*_eq_	
O1	1.24049 (15)	0.35130 (14)	0.11927 (4)	0.0235 (3)	
O2	1.36996 (17)	0.22076 (15)	0.15496 (6)	0.0312 (4)	
O3	1.43774 (19)	0.3126 (2)	0.09603 (6)	0.0416 (5)	
O4	0.97494 (15)	0.54091 (15)	0.30766 (5)	0.0278 (3)	
O5	0.43899 (16)	0.36705 (16)	0.49446 (6)	0.0332 (4)	
O6	0.7628 (3)	0.6180 (2)	0.45042 (6)	0.0531 (6)	
O7	0.7763 (2)	0.66683 (18)	0.52248 (6)	0.0458 (5)	
N1	1.35858 (19)	0.28882 (17)	0.12425 (6)	0.0266 (4)	
N2	0.64623 (18)	0.40002 (17)	0.47119 (6)	0.0259 (4)	
H2N	0.7079	0.3830	0.4518	0.031*	
C1	1.09373 (18)	0.41293 (17)	0.23173 (6)	0.0171 (3)	
H1A	1.1245	0.4667	0.2556	0.021*	
H1B	1.0903	0.3302	0.2437	0.021*	
C2	1.19274 (18)	0.41682 (17)	0.19389 (6)	0.0181 (3)	
H2A	1.2026	0.5006	0.1835	0.022*	
H2B	1.2796	0.3888	0.2045	0.022*	
C3	1.14771 (19)	0.33810 (17)	0.15614 (6)	0.0179 (3)	
H3	1.1464	0.2523	0.1659	0.021*	
C4	1.01167 (19)	0.37181 (18)	0.13737 (6)	0.0192 (3)	
C5	0.91408 (18)	0.37368 (16)	0.17714 (6)	0.0162 (3)	
H5	0.9131	0.2891	0.1883	0.019*	
C6	0.77159 (19)	0.39889 (18)	0.16342 (6)	0.0208 (4)	
H6A	0.7604	0.4858	0.1575	0.025*	
H6B	0.7510	0.3544	0.1360	0.025*	
C7	0.67731 (19)	0.35989 (18)	0.20001 (6)	0.0210 (4)	
H7A	0.6826	0.2715	0.2033	0.025*	
H7B	0.5863	0.3800	0.1910	0.025*	
C8	0.70534 (17)	0.41850 (16)	0.24500 (6)	0.0152 (3)	
C9	0.85615 (17)	0.41574 (16)	0.25560 (6)	0.0145 (3)	
H9	0.8759	0.3294	0.2618	0.017*	
C10	0.95365 (18)	0.45125 (15)	0.21774 (5)	0.0146 (3)	
C11	0.87786 (18)	0.47971 (17)	0.29951 (6)	0.0180 (3)	
C12	0.77583 (18)	0.46438 (17)	0.33337 (6)	0.0178 (3)	
H12	0.7923	0.4972	0.3617	0.021*	
C13	0.66101 (17)	0.40716 (16)	0.32705 (6)	0.0160 (3)	
C14	0.63076 (18)	0.34767 (16)	0.28289 (6)	0.0159 (3)	
C15	0.48044 (18)	0.34403 (18)	0.27359 (6)	0.0197 (4)	
H15A	0.4530	0.4229	0.2616	0.024*	
H15B	0.4631	0.2828	0.2507	0.024*	
C16	0.39605 (19)	0.31545 (18)	0.31388 (7)	0.0217 (4)	
H16A	0.4151	0.2324	0.3237	0.026*	
H16B	0.3020	0.3192	0.3055	0.026*	
C17	0.42072 (18)	0.40218 (18)	0.35212 (7)	0.0200 (4)	
C18	0.56722 (18)	0.39382 (17)	0.36569 (6)	0.0178 (3)	
H18	0.5851	0.4618	0.3863	0.021*	
C19	0.5989 (2)	0.27590 (18)	0.39060 (6)	0.0212 (4)	
H19A	0.5889	0.2075	0.3700	0.025*	
H19B	0.6921	0.2781	0.4001	0.025*	
C20	0.5117 (2)	0.25336 (19)	0.43106 (7)	0.0242 (4)	
C21	0.3676 (2)	0.2538 (2)	0.41571 (8)	0.0278 (4)	
H21A	0.3525	0.1853	0.3956	0.033*	
H21B	0.3090	0.2437	0.4415	0.033*	
C22	0.3332 (2)	0.3711 (2)	0.39203 (7)	0.0262 (4)	
H22A	0.3390	0.4378	0.4135	0.031*	
H22B	0.2407	0.3664	0.3820	0.031*	
C23	0.9709 (2)	0.2695 (2)	0.10587 (7)	0.0273 (4)	
H23A	1.0442	0.2508	0.0861	0.041*	
H23B	0.8944	0.2949	0.0885	0.041*	
H23C	0.9481	0.1979	0.1230	0.041*	
C24	1.0178 (2)	0.4897 (2)	0.11115 (7)	0.0269 (4)	
H24A	1.0641	0.5508	0.1285	0.040*	
H24B	0.9282	0.5174	0.1048	0.040*	
H24C	1.0649	0.4761	0.0835	0.040*	
C25	0.9541 (2)	0.58783 (17)	0.20873 (6)	0.0210 (4)	
H25A	0.9410	0.6313	0.2364	0.032*	
H25B	0.8829	0.6078	0.1882	0.032*	
H25C	1.0388	0.6111	0.1958	0.032*	
C26	0.6570 (2)	0.54965 (17)	0.24300 (7)	0.0209 (4)	
H26A	0.6996	0.5909	0.2183	0.031*	
H26B	0.6793	0.5906	0.2706	0.031*	
H26C	0.5614	0.5508	0.2389	0.031*	
C27	0.6788 (2)	0.21528 (16)	0.28652 (7)	0.0207 (4)	
H27A	0.7626	0.2129	0.3025	0.031*	
H27B	0.6910	0.1819	0.2569	0.031*	
H27C	0.6131	0.1676	0.3024	0.031*	
C28	0.3869 (2)	0.53116 (18)	0.33834 (7)	0.0234 (4)	
H28A	0.3980	0.5851	0.3636	0.035*	
H28B	0.2955	0.5344	0.3281	0.035*	
H28C	0.4457	0.5564	0.3145	0.035*	
C29	0.5290 (2)	0.34592 (19)	0.46787 (7)	0.0241 (4)	
C30	0.5474 (2)	0.1303 (2)	0.45119 (8)	0.0312 (5)	
H30A	0.4898	0.1139	0.4763	0.047*	
H30B	0.6393	0.1314	0.4610	0.047*	
H30C	0.5359	0.0673	0.4290	0.047*	
C31	0.6742 (2)	0.4853 (2)	0.50557 (7)	0.0288 (4)	
H31A	0.7311	0.4468	0.5279	0.035*	
H31B	0.5908	0.5086	0.5201	0.035*	
C32	0.7418 (3)	0.5969 (2)	0.48836 (8)	0.0343 (5)	
C33	0.8405 (4)	0.7825 (3)	0.51337 (11)	0.0540 (8)	
H33A	0.8960	0.7756	0.4867	0.065*	
H33B	0.8976	0.8052	0.5384	0.065*	
C34	0.7380 (4)	0.8759 (3)	0.50655 (12)	0.0644 (10)	
H34A	0.6815	0.8527	0.4818	0.097*	
H34B	0.7803	0.9530	0.5001	0.097*	
H34C	0.6846	0.8835	0.5333	0.097*	
O8	0.38763 (16)	0.1963 (2)	0.55652 (6)	0.0416 (5)	
H8	0.4016	0.2506	0.5379	0.062*	
C35	0.5070 (2)	0.1671 (3)	0.57816 (8)	0.0371 (5)	
H35A	0.5336	0.2342	0.5970	0.056*	
H35B	0.5757	0.1518	0.5562	0.056*	
H35C	0.4941	0.0950	0.5962	0.056*	

### Atomic displacement parameters (Å^2^)

**Table:**

	*U*^11^	*U*^22^	*U*^33^	*U*^12^	*U*^13^	*U*^23^
O1	0.0232 (7)	0.0269 (7)	0.0203 (6)	0.0070 (6)	0.0020 (5)	0.0015 (5)
O2	0.0276 (8)	0.0276 (7)	0.0384 (8)	0.0093 (7)	0.0014 (7)	0.0064 (7)
O3	0.0313 (9)	0.0535 (12)	0.0399 (9)	0.0083 (9)	0.0150 (8)	0.0041 (9)
O4	0.0220 (7)	0.0355 (8)	0.0259 (7)	−0.0126 (7)	0.0030 (6)	−0.0124 (6)
O5	0.0256 (7)	0.0384 (9)	0.0356 (8)	0.0058 (7)	0.0138 (7)	0.0080 (7)
O6	0.0758 (15)	0.0490 (11)	0.0345 (9)	−0.0191 (12)	0.0162 (10)	0.0097 (8)
O7	0.0582 (13)	0.0365 (9)	0.0427 (10)	−0.0103 (9)	0.0196 (9)	−0.0067 (8)
N1	0.0239 (8)	0.0255 (8)	0.0305 (9)	0.0041 (7)	0.0031 (7)	−0.0032 (7)
N2	0.0236 (8)	0.0304 (9)	0.0238 (8)	0.0012 (7)	0.0078 (7)	0.0030 (7)
C1	0.0158 (7)	0.0190 (8)	0.0166 (7)	−0.0007 (7)	−0.0027 (6)	0.0005 (6)
C2	0.0171 (8)	0.0192 (8)	0.0181 (7)	−0.0003 (7)	−0.0011 (6)	−0.0013 (6)
C3	0.0190 (8)	0.0178 (7)	0.0169 (7)	0.0040 (7)	−0.0012 (7)	−0.0005 (6)
C4	0.0205 (8)	0.0216 (8)	0.0156 (7)	0.0025 (7)	−0.0044 (7)	−0.0018 (6)
C5	0.0180 (8)	0.0153 (7)	0.0153 (7)	0.0008 (6)	−0.0040 (6)	−0.0027 (6)
C6	0.0185 (8)	0.0246 (9)	0.0192 (8)	0.0032 (7)	−0.0056 (7)	−0.0002 (7)
C7	0.0174 (8)	0.0230 (9)	0.0226 (8)	−0.0012 (7)	−0.0072 (7)	−0.0036 (7)
C8	0.0131 (7)	0.0136 (7)	0.0188 (7)	0.0003 (6)	−0.0042 (6)	−0.0010 (6)
C9	0.0135 (7)	0.0136 (7)	0.0164 (7)	−0.0016 (6)	−0.0027 (6)	−0.0010 (6)
C10	0.0163 (7)	0.0121 (7)	0.0153 (7)	−0.0001 (6)	−0.0026 (6)	−0.0003 (6)
C11	0.0161 (8)	0.0189 (8)	0.0192 (8)	−0.0019 (7)	−0.0014 (6)	−0.0027 (6)
C12	0.0161 (8)	0.0187 (8)	0.0184 (7)	−0.0010 (7)	−0.0017 (6)	−0.0007 (6)
C13	0.0132 (7)	0.0136 (7)	0.0213 (8)	0.0013 (6)	−0.0020 (6)	0.0025 (6)
C14	0.0132 (7)	0.0123 (7)	0.0221 (8)	−0.0003 (6)	−0.0034 (6)	0.0003 (6)
C15	0.0138 (8)	0.0183 (8)	0.0270 (9)	−0.0022 (7)	−0.0068 (7)	−0.0003 (7)
C16	0.0136 (8)	0.0190 (8)	0.0324 (10)	−0.0016 (7)	−0.0039 (7)	0.0018 (7)
C17	0.0128 (7)	0.0185 (8)	0.0286 (9)	−0.0001 (6)	−0.0002 (7)	0.0041 (7)
C18	0.0127 (7)	0.0181 (8)	0.0224 (8)	0.0001 (6)	−0.0007 (6)	0.0029 (7)
C19	0.0171 (8)	0.0215 (8)	0.0251 (9)	0.0029 (7)	0.0010 (7)	0.0056 (7)
C20	0.0204 (9)	0.0222 (9)	0.0300 (10)	0.0016 (8)	0.0051 (8)	0.0098 (8)
C21	0.0189 (9)	0.0272 (10)	0.0374 (11)	−0.0037 (8)	0.0045 (8)	0.0112 (9)
C22	0.0146 (8)	0.0294 (10)	0.0346 (10)	0.0004 (8)	0.0036 (8)	0.0086 (9)
C23	0.0278 (10)	0.0332 (11)	0.0209 (8)	0.0019 (9)	−0.0054 (8)	−0.0098 (8)
C24	0.0278 (10)	0.0320 (10)	0.0208 (9)	0.0063 (9)	−0.0019 (8)	0.0072 (8)
C25	0.0241 (9)	0.0130 (7)	0.0260 (9)	−0.0010 (7)	0.0022 (7)	−0.0001 (7)
C26	0.0195 (8)	0.0165 (8)	0.0268 (9)	0.0050 (7)	−0.0015 (7)	0.0041 (7)
C27	0.0185 (8)	0.0131 (7)	0.0306 (9)	0.0001 (7)	−0.0015 (7)	0.0015 (7)
C28	0.0178 (8)	0.0200 (8)	0.0323 (10)	0.0023 (7)	−0.0011 (8)	0.0037 (7)
C29	0.0208 (9)	0.0240 (9)	0.0273 (9)	0.0051 (8)	0.0054 (8)	0.0114 (8)
C30	0.0324 (11)	0.0251 (10)	0.0362 (11)	0.0044 (9)	0.0045 (10)	0.0124 (9)
C31	0.0334 (11)	0.0295 (10)	0.0233 (9)	0.0013 (9)	0.0068 (9)	0.0064 (8)
C32	0.0373 (12)	0.0313 (11)	0.0343 (11)	−0.0010 (10)	0.0121 (10)	0.0040 (9)
C33	0.0566 (19)	0.0508 (17)	0.0546 (17)	−0.0206 (16)	0.0115 (15)	−0.0036 (14)
C34	0.079 (2)	0.0497 (18)	0.064 (2)	−0.0230 (19)	−0.014 (2)	0.0164 (16)
O8	0.0170 (7)	0.0702 (14)	0.0375 (9)	−0.0064 (8)	−0.0026 (7)	0.0239 (9)
C35	0.0225 (10)	0.0544 (15)	0.0344 (11)	−0.0013 (11)	−0.0057 (9)	0.0076 (11)

### Geometric parameters (Å, º)

**Table:**

O1—N1	1.395 (2)	C16—H16B	0.9900
O1—C3	1.472 (2)	C17—C28	1.534 (3)
O2—N1	1.207 (2)	C17—C22	1.543 (3)
O3—N1	1.205 (3)	C17—C18	1.547 (3)
O4—C11	1.224 (2)	C18—C19	1.549 (3)
O5—C29	1.243 (3)	C18—H18	1.0000
O6—C32	1.196 (3)	C19—C20	1.536 (3)
O7—C32	1.343 (3)	C19—H19A	0.9900
O7—C33	1.469 (4)	C19—H19B	0.9900
N2—C29	1.338 (3)	C20—C29	1.531 (3)
N2—C31	1.440 (3)	C20—C21	1.537 (3)
N2—H2N	0.8800	C20—C30	1.543 (3)
C1—C2	1.528 (3)	C21—C22	1.531 (3)
C1—C10	1.545 (3)	C21—H21A	0.9900
C1—H1A	0.9900	C21—H21B	0.9900
C1—H1B	0.9900	C22—H22A	0.9900
C2—C3	1.514 (3)	C22—H22B	0.9900
C2—H2A	0.9900	C23—H23A	0.9800
C2—H2B	0.9900	C23—H23B	0.9800
C3—C4	1.542 (3)	C23—H23C	0.9800
C3—H3	1.0000	C24—H24A	0.9800
C4—C24	1.536 (3)	C24—H24B	0.9800
C4—C23	1.544 (3)	C24—H24C	0.9800
C4—C5	1.563 (3)	C25—H25A	0.9800
C5—C6	1.532 (3)	C25—H25B	0.9800
C5—C10	1.559 (2)	C25—H25C	0.9800
C5—H5	1.0000	C26—H26A	0.9800
C6—C7	1.530 (3)	C26—H26B	0.9800
C6—H6A	0.9900	C26—H26C	0.9800
C6—H6B	0.9900	C27—H27A	0.9800
C7—C8	1.541 (2)	C27—H27B	0.9800
C7—H7A	0.9900	C27—H27C	0.9800
C7—H7B	0.9900	C28—H28A	0.9800
C8—C26	1.541 (3)	C28—H28B	0.9800
C8—C9	1.566 (2)	C28—H28C	0.9800
C8—C14	1.588 (3)	C30—H30A	0.9800
C9—C11	1.528 (2)	C30—H30B	0.9800
C9—C10	1.569 (2)	C30—H30C	0.9800
C9—H9	1.0000	C31—C32	1.513 (3)
C10—C25	1.544 (2)	C31—H31A	0.9900
C11—C12	1.470 (3)	C31—H31B	0.9900
C12—C13	1.343 (2)	C33—C34	1.486 (6)
C12—H12	0.9500	C33—H33A	0.9900
C13—C18	1.519 (3)	C33—H33B	0.9900
C13—C14	1.527 (3)	C34—H34A	0.9800
C14—C15	1.554 (3)	C34—H34B	0.9800
C14—C27	1.556 (2)	C34—H34C	0.9800
C15—C16	1.528 (3)	O8—C35	1.417 (3)
C15—H15A	0.9900	O8—H8	0.8400
C15—H15B	0.9900	C35—H35A	0.9800
C16—C17	1.531 (3)	C35—H35B	0.9800
C16—H16A	0.9900	C35—H35C	0.9800
			
N1—O1—C3	114.75 (14)	C13—C18—C19	109.26 (15)
C32—O7—C33	118.6 (2)	C17—C18—C19	112.42 (15)
O3—N1—O2	128.6 (2)	C13—C18—H18	107.3
O3—N1—O1	112.79 (18)	C17—C18—H18	107.3
O2—N1—O1	118.61 (17)	C19—C18—H18	107.3
C29—N2—C31	121.79 (18)	C20—C19—C18	114.16 (16)
C29—N2—H2N	119.1	C20—C19—H19A	108.7
C31—N2—H2N	119.1	C18—C19—H19A	108.7
C2—C1—C10	113.04 (14)	C20—C19—H19B	108.7
C2—C1—H1A	109.0	C18—C19—H19B	108.7
C10—C1—H1A	109.0	H19A—C19—H19B	107.6
C2—C1—H1B	109.0	C29—C20—C19	114.11 (17)
C10—C1—H1B	109.0	C29—C20—C21	109.22 (18)
H1A—C1—H1B	107.8	C19—C20—C21	107.84 (17)
C3—C2—C1	110.78 (15)	C29—C20—C30	106.28 (17)
C3—C2—H2A	109.5	C19—C20—C30	109.05 (18)
C1—C2—H2A	109.5	C21—C20—C30	110.33 (18)
C3—C2—H2B	109.5	C22—C21—C20	111.27 (17)
C1—C2—H2B	109.5	C22—C21—H21A	109.4
H2A—C2—H2B	108.1	C20—C21—H21A	109.4
O1—C3—C2	108.99 (15)	C22—C21—H21B	109.4
O1—C3—C4	105.56 (14)	C20—C21—H21B	109.4
C2—C3—C4	114.24 (15)	H21A—C21—H21B	108.0
O1—C3—H3	109.3	C21—C22—C17	115.40 (18)
C2—C3—H3	109.3	C21—C22—H22A	108.4
C4—C3—H3	109.3	C17—C22—H22A	108.4
C24—C4—C3	111.32 (17)	C21—C22—H22B	108.4
C24—C4—C23	108.59 (16)	C17—C22—H22B	108.4
C3—C4—C23	106.91 (16)	H22A—C22—H22B	107.5
C24—C4—C5	114.53 (16)	C4—C23—H23A	109.5
C3—C4—C5	106.60 (14)	C4—C23—H23B	109.5
C23—C4—C5	108.59 (16)	H23A—C23—H23B	109.5
C6—C5—C10	110.96 (15)	C4—C23—H23C	109.5
C6—C5—C4	113.04 (14)	H23A—C23—H23C	109.5
C10—C5—C4	117.11 (15)	H23B—C23—H23C	109.5
C6—C5—H5	104.8	C4—C24—H24A	109.5
C10—C5—H5	104.8	C4—C24—H24B	109.5
C4—C5—H5	104.8	H24A—C24—H24B	109.5
C7—C6—C5	109.99 (15)	C4—C24—H24C	109.5
C7—C6—H6A	109.7	H24A—C24—H24C	109.5
C5—C6—H6A	109.7	H24B—C24—H24C	109.5
C7—C6—H6B	109.7	C10—C25—H25A	109.5
C5—C6—H6B	109.7	C10—C25—H25B	109.5
H6A—C6—H6B	108.2	H25A—C25—H25B	109.5
C6—C7—C8	114.13 (16)	C10—C25—H25C	109.5
C6—C7—H7A	108.7	H25A—C25—H25C	109.5
C8—C7—H7A	108.7	H25B—C25—H25C	109.5
C6—C7—H7B	108.7	C8—C26—H26A	109.5
C8—C7—H7B	108.7	C8—C26—H26B	109.5
H7A—C7—H7B	107.6	H26A—C26—H26B	109.5
C26—C8—C7	107.86 (15)	C8—C26—H26C	109.5
C26—C8—C9	109.78 (15)	H26A—C26—H26C	109.5
C7—C8—C9	110.79 (15)	H26B—C26—H26C	109.5
C26—C8—C14	110.28 (14)	C14—C27—H27A	109.5
C7—C8—C14	110.18 (14)	C14—C27—H27B	109.5
C9—C8—C14	107.95 (14)	H27A—C27—H27B	109.5
C11—C9—C8	108.15 (14)	C14—C27—H27C	109.5
C11—C9—C10	115.58 (14)	H27A—C27—H27C	109.5
C8—C9—C10	117.53 (14)	H27B—C27—H27C	109.5
C11—C9—H9	104.7	C17—C28—H28A	109.5
C8—C9—H9	104.7	C17—C28—H28B	109.5
C10—C9—H9	104.7	H28A—C28—H28B	109.5
C25—C10—C1	108.51 (15)	C17—C28—H28C	109.5
C25—C10—C5	113.92 (14)	H28A—C28—H28C	109.5
C1—C10—C5	107.61 (14)	H28B—C28—H28C	109.5
C25—C10—C9	112.32 (15)	O5—C29—N2	121.4 (2)
C1—C10—C9	108.08 (13)	O5—C29—C20	121.2 (2)
C5—C10—C9	106.15 (14)	N2—C29—C20	117.40 (18)
O4—C11—C12	119.42 (17)	C20—C30—H30A	109.5
O4—C11—C9	123.50 (17)	C20—C30—H30B	109.5
C12—C11—C9	117.08 (15)	H30A—C30—H30B	109.5
C13—C12—C11	124.57 (17)	C20—C30—H30C	109.5
C13—C12—H12	117.7	H30A—C30—H30C	109.5
C11—C12—H12	117.7	H30B—C30—H30C	109.5
C12—C13—C18	118.73 (16)	N2—C31—C32	112.32 (18)
C12—C13—C14	120.39 (16)	N2—C31—H31A	109.1
C18—C13—C14	120.69 (15)	C32—C31—H31A	109.1
C13—C14—C15	111.65 (15)	N2—C31—H31B	109.1
C13—C14—C27	106.56 (15)	C32—C31—H31B	109.1
C15—C14—C27	107.25 (15)	H31A—C31—H31B	107.9
C13—C14—C8	109.02 (14)	O6—C32—O7	125.7 (2)
C15—C14—C8	110.50 (15)	O6—C32—C31	125.2 (2)
C27—C14—C8	111.82 (15)	O7—C32—C31	109.10 (19)
C16—C15—C14	114.29 (16)	O7—C33—C34	109.2 (3)
C16—C15—H15A	108.7	O7—C33—H33A	109.8
C14—C15—H15A	108.7	C34—C33—H33A	109.8
C16—C15—H15B	108.7	O7—C33—H33B	109.8
C14—C15—H15B	108.7	C34—C33—H33B	109.8
H15A—C15—H15B	107.6	H33A—C33—H33B	108.3
C15—C16—C17	112.65 (16)	C33—C34—H34A	109.5
C15—C16—H16A	109.1	C33—C34—H34B	109.5
C17—C16—H16A	109.1	H34A—C34—H34B	109.5
C15—C16—H16B	109.1	C33—C34—H34C	109.5
C17—C16—H16B	109.1	H34A—C34—H34C	109.5
H16A—C16—H16B	107.8	H34B—C34—H34C	109.5
C16—C17—C28	110.23 (16)	C35—O8—H8	109.5
C16—C17—C22	111.17 (16)	O8—C35—H35A	109.5
C28—C17—C22	107.16 (17)	O8—C35—H35B	109.5
C16—C17—C18	108.77 (16)	H35A—C35—H35B	109.5
C28—C17—C18	110.14 (16)	O8—C35—H35C	109.5
C22—C17—C18	109.36 (16)	H35A—C35—H35C	109.5
C13—C18—C17	113.05 (15)	H35B—C35—H35C	109.5
			
C3—O1—N1—O3	−171.98 (18)	C12—C13—C14—C27	91.53 (19)
C3—O1—N1—O2	7.6 (3)	C18—C13—C14—C27	−83.32 (19)
C10—C1—C2—C3	−57.2 (2)	C12—C13—C14—C8	−29.3 (2)
N1—O1—C3—C2	77.22 (19)	C18—C13—C14—C8	155.85 (15)
N1—O1—C3—C4	−159.65 (15)	C26—C8—C14—C13	−61.40 (18)
C1—C2—C3—O1	175.89 (14)	C7—C8—C14—C13	179.64 (15)
C1—C2—C3—C4	58.1 (2)	C9—C8—C14—C13	58.54 (18)
O1—C3—C4—C24	−47.73 (19)	C26—C8—C14—C15	61.66 (19)
C2—C3—C4—C24	72.0 (2)	C7—C8—C14—C15	−57.31 (19)
O1—C3—C4—C23	70.72 (19)	C9—C8—C14—C15	−178.41 (15)
C2—C3—C4—C23	−169.56 (16)	C26—C8—C14—C27	−178.96 (15)
O1—C3—C4—C5	−173.29 (14)	C7—C8—C14—C27	62.08 (18)
C2—C3—C4—C5	−53.6 (2)	C9—C8—C14—C27	−59.02 (18)
C24—C4—C5—C6	59.7 (2)	C13—C14—C15—C16	−39.6 (2)
C3—C4—C5—C6	−176.67 (16)	C27—C14—C15—C16	76.7 (2)
C23—C4—C5—C6	−61.8 (2)	C8—C14—C15—C16	−161.15 (15)
C24—C4—C5—C10	−71.1 (2)	C14—C15—C16—C17	55.7 (2)
C3—C4—C5—C10	52.5 (2)	C15—C16—C17—C28	60.9 (2)
C23—C4—C5—C10	167.32 (16)	C15—C16—C17—C22	179.60 (16)
C10—C5—C6—C7	−64.4 (2)	C15—C16—C17—C18	−59.9 (2)
C4—C5—C6—C7	161.69 (16)	C12—C13—C18—C17	144.52 (17)
C5—C6—C7—C8	56.1 (2)	C14—C13—C18—C17	−40.5 (2)
C6—C7—C8—C26	75.1 (2)	C12—C13—C18—C19	−89.5 (2)
C6—C7—C8—C9	−45.1 (2)	C14—C13—C18—C19	85.45 (19)
C6—C7—C8—C14	−164.45 (15)	C16—C17—C18—C13	51.0 (2)
C26—C8—C9—C11	58.58 (18)	C28—C17—C18—C13	−69.9 (2)
C7—C8—C9—C11	177.61 (14)	C22—C17—C18—C13	172.58 (16)
C14—C8—C9—C11	−61.66 (18)	C16—C17—C18—C19	−73.29 (19)
C26—C8—C9—C10	−74.52 (19)	C28—C17—C18—C19	165.80 (16)
C7—C8—C9—C10	44.5 (2)	C22—C17—C18—C19	48.3 (2)
C14—C8—C9—C10	165.23 (14)	C13—C18—C19—C20	179.00 (16)
C2—C1—C10—C25	−70.77 (19)	C17—C18—C19—C20	−54.6 (2)
C2—C1—C10—C5	52.94 (19)	C18—C19—C20—C29	−64.7 (2)
C2—C1—C10—C9	167.18 (14)	C18—C19—C20—C21	56.9 (2)
C6—C5—C10—C25	−64.3 (2)	C18—C19—C20—C30	176.67 (18)
C4—C5—C10—C25	67.5 (2)	C29—C20—C21—C22	68.4 (2)
C6—C5—C10—C1	175.38 (15)	C19—C20—C21—C22	−56.1 (2)
C4—C5—C10—C1	−52.79 (19)	C30—C20—C21—C22	−175.09 (19)
C6—C5—C10—C9	59.85 (18)	C20—C21—C22—C17	56.4 (3)
C4—C5—C10—C9	−168.32 (15)	C16—C17—C22—C21	69.3 (2)
C11—C9—C10—C25	−55.9 (2)	C28—C17—C22—C21	−170.23 (18)
C8—C9—C10—C25	73.8 (2)	C18—C17—C22—C21	−50.9 (2)
C11—C9—C10—C1	63.80 (19)	C31—N2—C29—O5	0.0 (3)
C8—C9—C10—C1	−166.48 (15)	C31—N2—C29—C20	−177.81 (18)
C11—C9—C10—C5	179.00 (15)	C19—C20—C29—O5	154.10 (19)
C8—C9—C10—C5	−51.27 (19)	C21—C20—C29—O5	33.3 (2)
C8—C9—C11—O4	−144.77 (19)	C30—C20—C29—O5	−85.7 (2)
C10—C9—C11—O4	−10.6 (3)	C19—C20—C29—N2	−28.1 (3)
C8—C9—C11—C12	35.9 (2)	C21—C20—C29—N2	−148.88 (18)
C10—C9—C11—C12	170.02 (16)	C30—C20—C29—N2	92.1 (2)
O4—C11—C12—C13	174.69 (19)	C29—N2—C31—C32	−134.1 (2)
C9—C11—C12—C13	−5.9 (3)	C33—O7—C32—O6	2.8 (4)
C11—C12—C13—C18	177.53 (17)	C33—O7—C32—C31	−178.3 (3)
C11—C12—C13—C14	2.6 (3)	N2—C31—C32—O6	4.9 (4)
C12—C13—C14—C15	−151.67 (17)	N2—C31—C32—O7	−174.1 (2)
C18—C13—C14—C15	33.5 (2)	C32—O7—C33—C34	87.1 (3)

### Hydrogen-bond geometry (Å, º)

**Table:**

*D*—H···*A*	*D*—H	H···*A*	*D*···*A*	*D*—H···*A*
N2—H2*N*···O8^i^	0.88	2.04	2.806 (3)	144
O8—H8···O5	0.84	1.89	2.728 (2)	177
C1—H1*A*···O4	0.99	2.34	2.968 (2)	120
C19—H19*B*···O8^i^	0.99	2.40	3.359 (3)	163
C25—H25*A*···O4	0.98	2.41	3.058 (3)	123
C34—H34*B*···O5^ii^	0.98	2.58	3.515 (4)	160

Symmetry codes: (i) *x*+1/2, −*y*+1/2, −*z*+1; (ii) *x*+1/2, −*y*+3/2, −*z*+1.
